# Clinical and systemic factors associated with pressure ulcer development: a retrospective evaluation of 339 patients in a palliative care unit

**DOI:** 10.3389/fmed.2025.1686611

**Published:** 2025-12-09

**Authors:** Fatih Oner Kaya, Selçuk Şimşek

**Affiliations:** 1 Department of Internal Medicine, Maltepe University Hospital, Istanbul, Türkiye; 2 Department of Palliative Medicine, Maltepe University Hospital, Istanbul, Türkiye

**Keywords:** pressure ulcer, palliative care, cardiovascular disease, stroke, Braden Scale, care dependency, risk factors, retrospective study

## Abstract

**Background:**

Pressure ulcers (PUs) remain a prevalent and serious complication in palliative care settings, often resulting from a combination of immobility, systemic illness, and inadequate preventive strategies. Understanding the multifactorial risk landscape is essential for developing effective interventions.

**Objective:**

This study aimed to determine the prevalence of pressure ulcers and to identify clinical and systemic risk factors associated with PU development in a palliative care population.

**Methods:**

A retrospective, single-center study was conducted by reviewing medical records of 339 adult patients hospitalized for more than 24 h in the palliative care unit of Maltepe University Hospital between June 2021 and November 2023. Demographic data, comorbidities, Braden and Care Dependency Scale (CDS) scores, and PU characteristics were analyzed. Multivariable logistic regression was used to identify independent risk and protective factors.

**Results:**

The overall prevalence of pressure ulcers was 33.6%. A total of 385 ulcers were recorded among 114 patients, with deep tissue injury (26%) being the most common type. Significant independent risk factors included stroke (OR: 4.89), gastrointestinal diseases (OR: 8.72), paraplegia (OR: 17.65), recent confusion (OR: 148.00), hypertension (OR: 2.98), coronary artery disease (OR: 3.42), arrhythmia (OR: 3.05), and heart failure (OR: 3.88). Longer hospital stay also increased PU risk (OR: 1.04 per day). In contrast, adherence to PU prevention protocols (OR: 0.18) and complete care dependency (OR: 0.02) were significant protective factors.

**Conclusion:**

Pressure ulcers are alarmingly common in palliative care patients and are closely linked to multiple comorbid conditions. Targeted preventive measures focusing on cardiovascular and neurological stability, nutritional support, cognitive function, and strict adherence to care protocols are vital. Proactive, multidisciplinary management and individualized risk assessment should be integrated into routine care to reduce PU burden and improve patient outcomes.

## Introduction

Pressure ulcers (PUs), also known as bedsores or decubitus ulcers, are tissue injuries resulting from prolonged exposure of the skin to moderate or high levels of pressure. This exposure impairs local blood flow and oxygen delivery, ultimately leading to hypoxia and ulceration due to decreased tissue perfusion (1, 2). These lesions can range from superficial erythema to deep wounds extending to muscle and bone tissue. Although most commonly found over bony prominences such as the heels, sacrum, hips, and elbows, they can develop on any area subjected to sustained pressure (3).

PUs represent a significant clinical issue, particularly in frail or immobile individuals, with a more pronounced impact in intensive care and palliative care settings that require advanced clinical support (4). Ulcer development reduces patients’ quality of life, increases healthcare costs, and complicates clinical management by introducing secondary infections and other complications (5).

Risk factors for PU development include immobility, impaired consciousness, poor nutritional status, and urinary/fecal incontinence. Additionally, chronic systemic conditions, especially cardiovascular comorbidities, are key contributing factors (6). Conditions such as hypertension, heart failure, and peripheral vascular disease impair tissue perfusion, causing hypoxia and compromising skin integrity (7).

Furthermore, malnutrition due to gastrointestinal diseases, paraplegia, post-stroke mobility loss, and recent episodes of confusion are also directly associated with PU development. In this context, identifying independent risk factors associated with PU development in patients admitted to palliative care units is critical for early recognition of high-risk individuals and optimization of preventive strategies.

This study aims to determine the prevalence of pressure ulcers in adult patients followed in a palliative care unit and to assess the relationship between ulcer development and comorbid cardiovascular, neurological, and gastrointestinal conditions.

Importantly, the loss of blood flow and oxygen delivery due to sustained pressure initiates local ischemia and necrosis, which create an optimal environment for microbial colonization. The resulting infection triggers acute inflammation in the surrounding viable tissue, worsening edema and oxygen diffusion and perpetuating the ischemia–inflammation cycle that drives ulcer progression (8, 9).

## Materials and methods

Study Design and Population this single-center, descriptive, and analytical retrospective study was conducted using medical records of patients admitted to the palliative care unit of a university hospital in Istanbul, Maltepe University, between June 2021 and November 2023. The objective was to evaluate potential risk factors associated with pressure ulcer (PU) development.

Participants and Inclusion Criteria The study included adult patients (aged 18 years and older) who were hospitalized in the palliative care unit for more than 24 h during the specified period. Patients who already had pressure ulcers at admission, those under 19 years old, and those with incomplete medical records were excluded.

### Data collection

Data were retrospectively retrieved from the hospital information system. Clinical, nursing, and assessment forms in each patient’s electronic file were reviewed. Demographic data, existing diagnoses, clinical observations, and risk assessment tool scores were recorded. The Braden Pressure Ulcer Risk Assessment Scale and Care Dependency Scale (CDS) scores were obtained from nursing admission forms. The stage, anatomical location, and total number of pressure ulcers were verified from clinical records.

### Assessment tools

- Braden Score: Used to assess the risk of pressure ulcer development, the Braden Scale includes six subscales: sensory perception, moisture, activity, mobility, nutrition, and friction/shear. The total score ranges from 6 to 23, with lower scores indicating higher risk.
- Care Dependency Scale (CDS): This tool assesses physical and psychosocial care dependency across 15 items, scored from 1 (fully dependent) to 5 (almost independent). Total scores range from 15 to 75, with lower scores indicating greater care dependency.

Definition and Classification of Pressure Ulcers Pressure ulcers were defined as localized damage to the skin and/or underlying tissue, typically over bony prominences, due to pressure or pressure combined with shear.

### Classification was based on the European pressure ulcer advisory panel (EPUAP) staging system

- Stage I: Intact skin with non-blanchable erythema
- Stage II: Partial-thickness skin loss with exposed dermis or fluid-filled blister
- Stage III: Full-thickness skin loss; subcutaneous fat may be visible, but not bone, tendon, or muscle
- Stage IV: Full-thickness tissue loss with exposed bone, tendon, or muscle

Ulcer locations were categorized anatomically (e.g., sacrum, heels, trochanter, ankles), and data on ulcer origin (in-unit, from another facility, or home), total ulcer count, infection status, and associated moisture lesions were also recorded.

### Ethical approval

Ethical approval for this study was granted by the Maltepe University GETAT Ethics Committee (Approval No: 2/2023; Date: 02.05.2023). All data were anonymized and handled in accordance with confidentiality principles.

### Statistical analysis

Data analysis was performed using IBM SPSS Statistics 26.0 (IBM Corp., Armonk, NY, USA). Continuous variables were summarized as mean ± standard deviation (SD) or median (minimum–maximum), and categorical variables were presented as frequencies and percentages.

Differences between categorical variables were evaluated using Pearson’s chi-square test or Fisher’s exact test. For continuous variables, independent samples *t*-tests were used for normally distributed data, while the Mann–Whitney U test was applied for non-normally distributed data.

To identify independent risk factors for PU development, multivariable logistic regression analysis was performed. Variables found to be statistically significant (*p* < 0.05) in univariate analysis were included in the model. Results were reported as odds ratios (OR) with 95% confidence intervals (CI).

A *p*-value of less than 0.05 was considered statistically significant for all analyses.

## Results

A total of 339 patients hospitalized in the palliative care unit were evaluated. As shown in Table 1, 61.5% of the participants were male, and the mean age was 54.8 ± 10.9 years. The overall prevalence of pressure ulcer (PU) development during follow-up was 33.6% (*n* = 114), while no ulcer development was observed in the remaining 66.4% (*n* = 225).

**Table 1:** Baseline characteristics of the study population.

Variable	Total (*n* = 339)
Age (years, mean ± SD)	54.8 ± 10.9
Gender—Male	208 (61.5%)
Gender—Female	131 (38.5%)
Length of hospital stay (median)	15 days [2–89]
Braden score (mean ± SD)	3.1 ± 1.2
CDS score (mean ± SD)	32.4 ± 6.7
Neurological diseases	172 (50.7%)
Cardiovascular diseases	183 (54.0%)
Gastrointestinal diseases	94 (27.7%)
Urinary system diseases	79 (23.3%)
Malnutrition diagnosed	121 (35.7%)
Recent confusion (last 7 days)	69 (20.4%)
Mobility status – Immobile	246 (72.6%)
PU prevention protocol followed	191 (56.3%)
PU developed during follow-up	114 (33.6%)

When all stages of pressure ulcers were considered, the prevalence was calculated as 33.6% [95% CI, 28.5–38.9]; this rate decreased to 31.3% [95% CI, 26.4–36.5] when Stage I ulcers were excluded. A total of 385 ulcers were recorded in 114 patients who developed PU. The most frequently observed type was deep tissue injury (26%), followed by unstageable ulcers (20.2%) and Stage IV ulcers (18.4%). The most affected anatomical regions were the sacrum, heels, trochanteric areas, and ankles.

As presented in Table 2, a comparison between patients with and without PU revealed statistically significant associations between PU development and the presence of stroke, paraplegia, gastrointestinal and urinary system diseases, as well as cardiovascular comorbidities such as hypertension, coronary artery disease (CAD), arrhythmia, and heart failure.

**Table 2:** Comparison of categorical variables between patients with and without pressure ulcers.

Variable	Total (*n* = 339)	Non-PU (*n* = 225)	PU (*n* = 114)	*p*-value	OR (95% CI)
Gender—Male	208 (61.5%)	134 (59.5%)	74 (64.9%)	0.29	1.26 (0.82–1.95)
Neurological disease	172 (50.7%)	96 (42.6%)	76 (66.7%)	<0.001	2.74 (1.69–4.43)
Cardiovascular disease	183 (54.0%)	104 (46.2%)	79 (69.3%)	<0.001	2.69 (1.64–4.41)
Gastrointestinal disease	94 (27.7%)	38 (16.9%)	56 (49.1%)	<0.001	4.83 (2.87–8.13)
Urinary system disease	79 (23.3%)	39 (17.3%)	40 (35.1%)	0.001	2.59 (1.55–4.34)
Paraplegia	41 (12.1%)	7 (3.1%)	34 (29.8%)	<0.001	13.95 (6.00–32.45)
Recent confusion (last 7 days)	69 (20.4%)	9 (4.0%)	60 (52.6%)	<0.001	26.3 (12.1–56.9)
PU prevention protocol followed	191 (56.3%)	150 (66.7%)	41 (36.0%)	<0.001	0.28 (0.18–0.45)
Fully dependent (CDS classification)	112 (33.0%)	52 (23.1%)	60 (52.6%)	<0.001	3.64 (2.26–5.86)

Management-related factors were also examined. Patients who experienced confusion in the last 7 days prior to ulcer development had a significantly increased risk of PU (52.6% vs. 4.0%, *p* < 0.001). In addition, Braden pressure ulcer risk scores were significantly lower in the PU group (mean: 2.2 ± 0.8 vs. 3.5 ± 1.1; *p* < 0.001), while Care Dependency Scale (CDS) scores were also markedly lower in the PU group, indicating higher care dependency.

As shown in Table 3, a significant difference was observed in median hospital stay between groups: 19 days [4–89] in the PU group and 12 days [2–44] in the non-PU group (*p* < 0.001). Similarly, the Braden and CDS scores also differed significantly between groups.

**Table 3:** Comparison of continuous variables between patients with and without pressure ulcers.

Variable	Total (*n* = 339)	Non-PU (*n* = 225)	PU (*n* = 114)	*p*-value
Age (years)	54.8 ± 10.9	53.9 ± 11.1	56.6 ± 10.3	0.08
Length of hospital stay (days)	15 [2–89]	12 [2–44]	19 [4–89]	<0.001
Braden score	3.1 ± 1.2	3.5 ± 1.1	2.2 ± 0.8	<0.001
CDS score	32.4 ± 6.7	35.1 ± 5.8	28.3 ± 5.9	<0.001

Detailed comparison of Braden subscale components is presented in Table 4. The PU group scored significantly lower across all six subcomponents: sensory perception, moisture, activity, mobility, nutrition, and friction/shear (*p* < 0.001 for all), indicating a higher clinical risk profile.

**Table 4:** Comparison of Braden Scale subdimensions between PU and non-PU groups.

Subscale	Non-PU (n = 225)	PU (n = 114)	*p*-value
Sensory perception	3.6 ± 0.5	2.1 ± 0.6	<0.001
Moisture	3.3 ± 0.6	2.0 ± 0.7	<0.001
Activity	3.5 ± 0.7	2.1 ± 0.5	<0.001
Mobility	3.4 ± 0.6	2.0 ± 0.6	<0.001
Nutrition	3.2 ± 0.5	2.1 ± 0.5	<0.001
Friction and shear	2.9 ± 0.3	1.8 ± 0.4	<0.001

As displayed in Table 5, the time distribution of PU onset shows that ulcers most commonly developed between days 3 and 11 of hospitalization, with a peak on day 11 (13.2%). The cumulative incidence by day 13 reached over 90%, suggesting early onset in most cases.

**Table 5:** Distribution of PU onset by day of hospital stay.

Day of PU onset	Frequency	Percentage (%)	Cumulative percentage (%)
2	4	5.9	5.9
3	8	11.8	17.7
4	6	8.8	26.5
5	5	7.4	33.9
6	4	5.9	39.8
7	8	11.8	51.6
8	6	8.8	60.4
9	5	7.4	67.8
11	9	13.2	81.0
12	3	4.4	85.4
13	4	5.9	91.3
18	2	2.9	94.2
19	2	2.9	97.1
21	1	1.5	98.6
25	1	1.5	100.1

Multivariable logistic regression analysis was performed to determine independent risk factors for PU development (see Table 6). Stroke (OR: 4.89), gastrointestinal disease (OR: 8.72), paraplegia (OR: 17.65), recent confusion (OR: 148.00), hypertension (OR: 2.98), CAD (OR: 3.42), arrhythmia (OR: 3.05), and heart failure (OR: 3.88) were found to be statistically significant risk factors. Notably, hospital length of stay was also an independent predictor; each additional day increased the risk of PU development by 4% (OR: 1.04, 95% CI: 1.02–1.07; *p* < 0.001). On the other hand, adherence to PU prevention protocols (OR: 0.18) and complete care dependency (OR: 0.02) were found to be protective.

**Table 6:** Multivariable logistic regression analysis of risk factors associated with PU development.

Variable	OR	95% CI	*p*-value
Stroke	4.89	2.51–9.52	<0.001
Gastrointestinal disease	8.72	4.21–18.08	<0.001
Paraplegia	17.65	6.91–45.12	<0.001
Recent confusion (last 7 days)	148.00	46.75–468.33	<0.001
Hypertension	2.98	1.47–6.04	0.002
Coronary artery disease	3.42	1.71–6.87	<0.001
Arrhythmia	3.05	1.47–6.33	0.003
Heart failure	3.88	1.88–8.01	<0.001
Length of hospital stay (per day)	1.04	1.02–1.07	<0.001
PU prevention protocol followed	0.18	0.11–0.30	<0.001
Fully dependent (CDS classification)	0.02	0.01–0.05	<0.001

**Figure 1:**
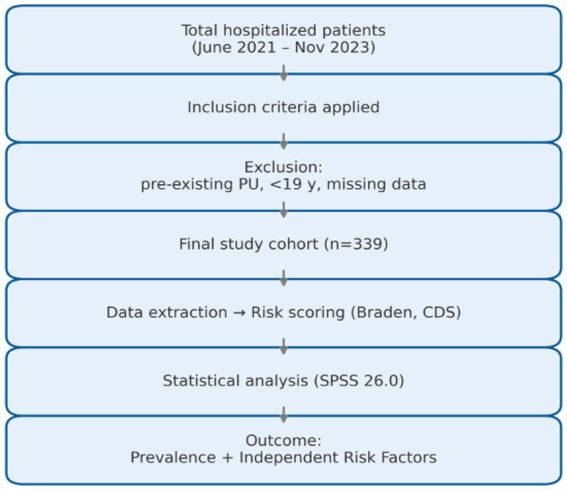
Flow diagram of study design and analysis pipeline. The overall study workflow is summarized in Figure 1, illustrating patient selection, inclusion and exclusion criteria, data extraction, and analytical stages.

The overall study design and patient selection process are summarized in Figure 1. Of the 482 patients initially screened, 143 were excluded due to pre-existing ulcers, incomplete medical data, or hospitalization shorter than 24 h. The remaining 339 patients met inclusion criteria and were subsequently analyzed. This flowchart provides a transparent overview of data collection, exclusion logic, and analytical stratification, ensuring reproducibility and methodological clarity.

**Figure 2:**
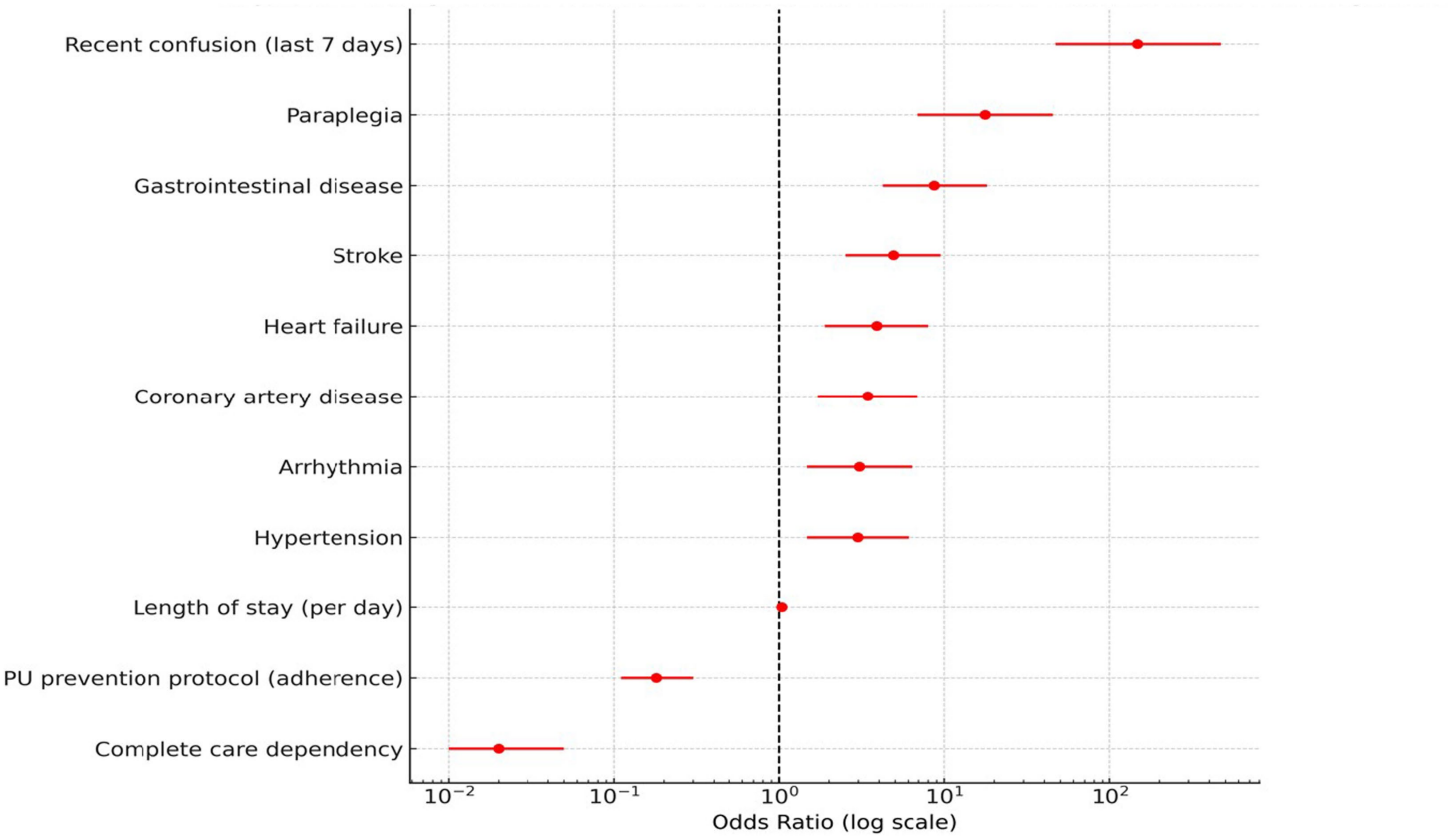
Independent risk and protective factors for pressure ulcer development. Summarizes the results of the multivariable logistic regression analysis, demonstrating the relative magnitude of independent risk and protective factors associated with pressure ulcer development.

As illustrated in Figure 2, the forest plot presents the multivariable logistic regression outcomes, visualizing the relative contribution of each independent predictor. Confusion in the preceding week (OR 148.00, *p* < 0.001) emerged as the most powerful risk factor, followed by paraplegia (OR 17.65) and gastrointestinal diseases (OR 8.72). Conversely, adherence to prevention protocols (OR 0.18) and complete care dependency (OR 0.02) demonstrated strong protective associations. This visual summary underscores the multifactorial etiology of pressure ulcers, integrating neurological, cardiovascular, and behavioral determinants into a single analytic framework.

**Figure 3:**
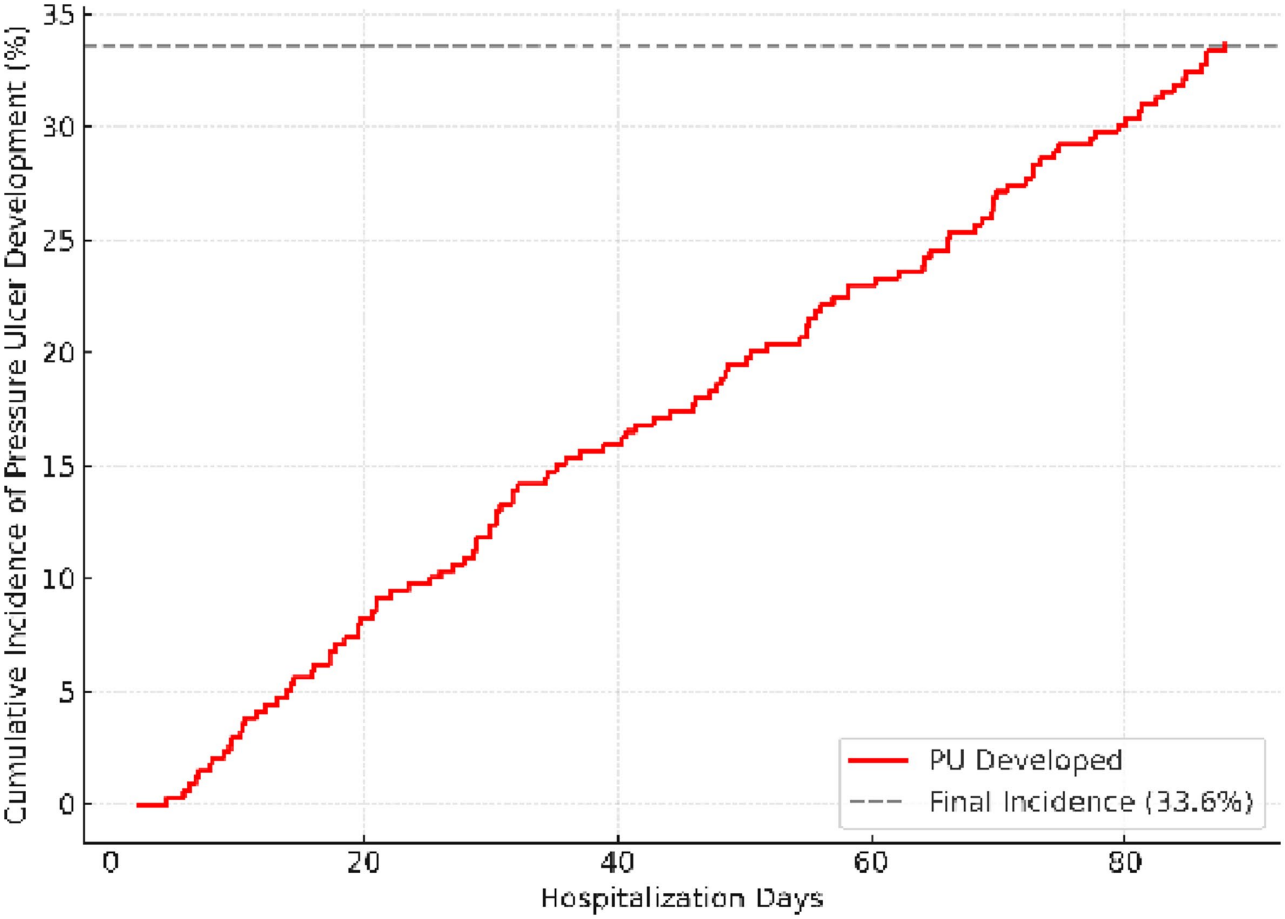
Kaplan–Meier curve illustrating cumulative incidence of pressure ulcer development during hospitalization. The Kaplan–Meier curve demonstrates cumulative incidence of ulcer development over-hospitalization days. The risk increased notably after the 10th day, highlighting the impact of prolonged immobility on PU onset.

Figure 3 demonstrates the cumulative incidence of pressure ulcer development over hospitalization time. The majority of ulcers appeared within the first 13 days of admission, with a steep rise between days 7 and 12. This pattern supports the hypothesis that immobility and inadequate early preventive care are pivotal in the pathogenesis of PUs. The early plateau phase observed after day 20 suggests that prevention strategies implemented beyond the second week may have limited efficacy once critical tissue damage has occurred.

**Figure 4:**
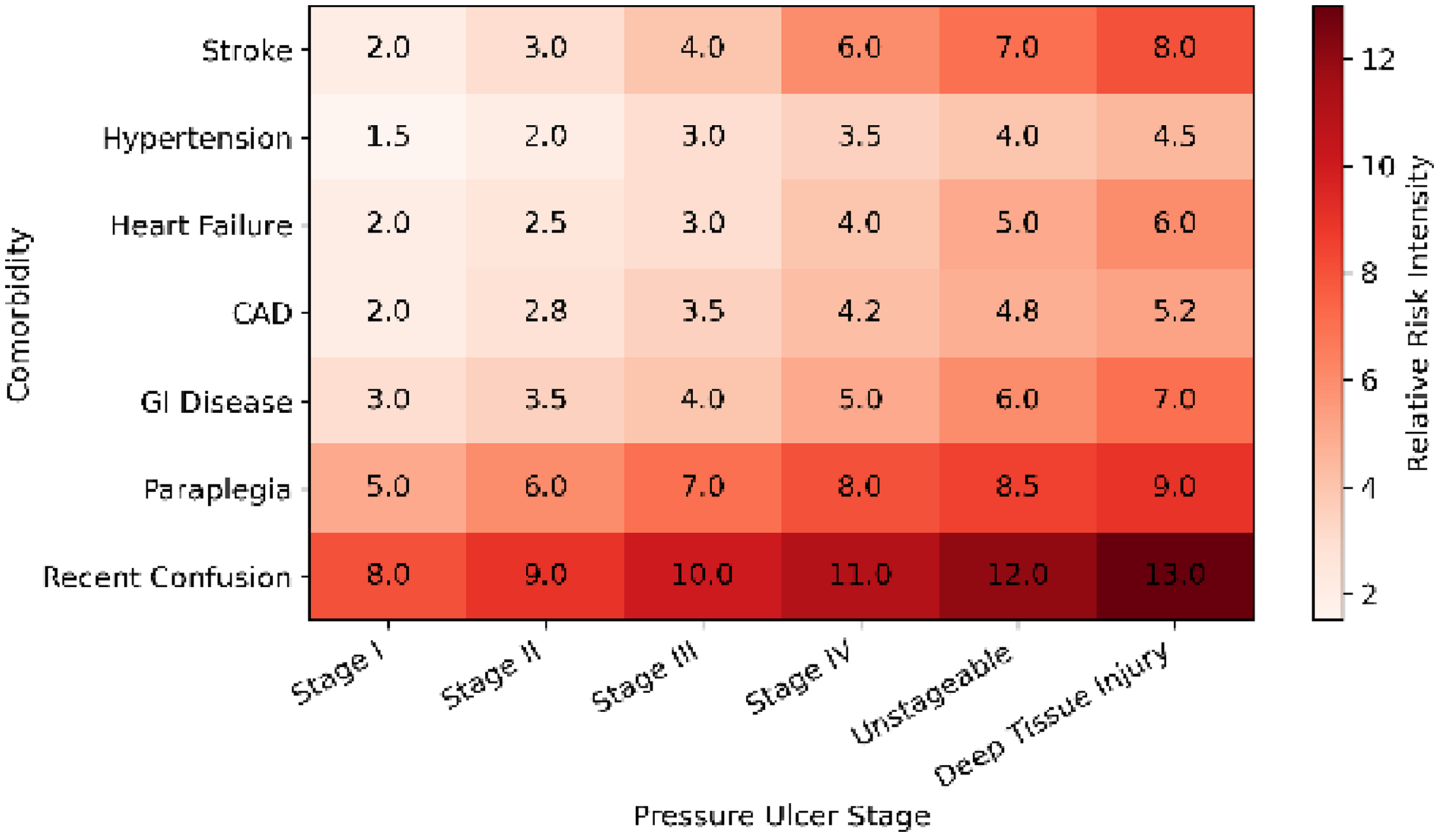
Heatmap depicting the relationship between comorbidities and pressure ulcer severity stages. The heatmap demonstrates clustering between advanced ulcer stages and major comorbidities such as stroke, paraplegia, and hypertension. Red intensity reflects higher odds ratios derived from multivariable modeling.

The comorbidity heatmap in Figure 4 provides a comprehensive visual representation of interrelations between systemic diseases and ulcer severity. The strongest clustering occurred between stroke, paraplegia, and hypertension, predominantly associated with Stage III–IV ulcers. This multidimensional analysis highlights the synergistic burden of neurological and cardiovascular dysfunctions, emphasizing the need for integrated monitoring of patients exhibiting overlapping comorbidities.

## Discussion

This study aimed to determine the prevalence of pressure ulcer (PU) development and the associated risk factors among patients followed in a palliative care unit. According to the results, the prevalence of PU was found to be 33.6%, which is relatively high when compared to similar patient populations.

The comprehensive visual analyses presented in Figures 1–4 provide a more intuitive understanding of how different systemic and neurological factors converge to influence ulcer development. Beyond numerical associations, these figures collectively illustrate the temporal evolution, clinical clustering, and protective mechanisms that shape the course of pressure ulcer formation in frail, palliative-care populations.

Multivariable logistic regression analysis revealed that stroke, gastrointestinal diseases, paraplegia, and confusion within the last 7 days were prominent neurological and systemic risk factors for PU development. Notably, cardiovascular comorbidities such as hypertension (OR: 2.98), coronary artery disease (OR: 3.42), arrhythmia (OR: 3.05), and heart failure (OR: 3.88) were identified as independent and significant risk factors. These findings underscore the role of impaired tissue perfusion in ulcer formation.

On the other hand, adherence to PU prevention guidelines (OR: 0.18) and total care dependency (OR: 0.02) were found to be significant protective factors against ulcer development. These results demonstrate that not only medical diagnoses but also the organization of care processes and adherence to guideline-based interventions play a crucial role in PU risk management.

The incidence of pressure ulcers in our palliative care unit was found to be notably high. In a systematic review by Ferris et al. involving 63,118 patients, the overall incidence was reported as 12.4%. When individual studies in the review are examined, the prevalence ranged between 9.9 and 54.7%, reflecting variability across different clinical settings and patient demographics (10).

The high prevalence in our study may be attributed to several factors, primarily that 227 out of 339 patients (66.9%) had identifiable risk factors for PU development, supporting the elevated rates observed.

Deep tissue injuries (26%) were the most common type of ulcer in our study, followed by unstageable ulcers (20.2%) and Stage IV ulcers (18.4%). This distribution suggests a delay in early identification and possibly inadequate implementation of preventive care. Anatomically, the most commonly affected areas were the sacrum, heels, trochanteric regions, and ankles—sites typically subjected to prolonged pressure due to immobility. Furthermore, many patients with PUs had a history of paraplegia or stroke, which corresponds to lower scores in Braden subscales such as mobility, activity, and sensory perception.

PU development is a serious complication that directly affects clinical monitoring and care quality, especially in post-stroke patients. In our study, the risk of PU was nearly 4.9 times higher in patients with a history of stroke (OR: 4.89), aligning with findings from the literature. Yu et al. (11), in their systematic review and meta-analysis, reported that PU prevalence increased significantly post-discharge in stroke patients, particularly in settings lacking home-based medical services.

The subgroup of stroke patients with coexisting paraplegia and sensory-motor deficits had significantly lower Braden scores, thereby increasing PU susceptibility. Therefore, a comprehensive, interdisciplinary monitoring approach for post-stroke patients is critical not only for rehabilitation but also for PU prevention (12).

As illustrated in Figure 4, the clustering of stroke, paraplegia, and hypertension reflects a compounded risk pattern, supporting the concept that neurological impairment combined with vascular dysfunction dramatically increases vulnerability to deep-tissue injury. This visual correlation parallels the statistical findings and adds a clinical dimension to their interpretation.

Figure 3 further complements this observation by revealing that nearly all ulcer events occurred within the first 2 weeks of hospitalization. The steep portion of the Kaplan–Meier curve between days 7 and 13 emphasizes the early critical window when preventive interventions are most impactful. Together, these visual summaries transform complex regression outputs into patterns that are immediately meaningful in bedside care.

Nutritional status is a key modifiable risk factor in the pathogenesis of pressure ulcers. Inadequate energy and protein intake compromises tissue integrity and delays wound healing. Malabsorption and inflammatory processes due to gastrointestinal diseases contribute to imbalances in macro- and micronutrient levels, impairing recovery.

Large-scale studies, including the National Pressure Ulcer Long-Term Care Study, have demonstrated significant associations between weight loss, poor dietary intake, and PU risk. Patients with gastrointestinal disorders such as Crohn’s disease, celiac disease, or pancreatitis should be closely monitored not only for medical treatment but also for nutritional status and weight loss (13, 14).

A systematic review and meta-analysis by Stratton et al. (15) showed that both oral and enteral nutritional support had protective effects in patients at risk of PU. This suggests that even in patients with compromised gastrointestinal function, appropriate enteral nutrition should be an integral part of PU prevention strategies.

Similarly, a study by Shahin et al. found that unintentional weight loss (5–10%) was strongly associated with PU development. Conducted in German long-term care settings, the study showed that PU incidence was significantly higher in undernourished elderly individuals, particularly those with multiple comorbidities.

The 2014 National Pressure Ulcer Consensus Conference also emphasized the central role of malnutrition and comorbidities in PU pathogenesis (16, 17). These findings highlight the necessity of regular nutritional assessment and monitoring of patients with gastrointestinal illnesses to reduce ulcer risk.

Another critical factor contributing to PU development is acute confusion. Impaired cognitive function limits patients’ ability to reposition themselves, recognize discomfort, or seek help in time. This can lead to the progression of undetected ulcers and increased complication risks. Moreover, caring for confused patients requires additional staffing and time, posing challenges for institutions with limited resources (18).

Our findings showed that adherence to PU prevention guidelines and higher levels of care dependency had protective effects. Interestingly, patients with moderate dependency showed a higher PU incidence, possibly due to being perceived as less vulnerable and therefore receiving less preventive care. This phenomenon is often seen in palliative patients who are immobile but still communicative. Our results parallel findings by De Laat et al. (19) who reported that previous PU history in paraplegic patients did not necessarily correlate with future care compliance.

Structured, continuous, and individualized nursing interventions can significantly reduce PU risk in patients with limited self-care capacity. The literature shows that self-management- based interventions are effective not only in knowledge transfer but also in encouraging behavioral changes in high-risk individuals (20). Our findings suggest that patients appearing functionally independent may still require intensive preventive care and should not be overlooked.

The association between cardiovascular diseases and PU development in our study aligns with existing literature. In a pivotal study where perfusion disorders, mechanical ventilation, and surgical interventions were identified as PU predictors, all three variables were statistically significant (*p* < 0.001). Cardiovascular dysfunction—often seen in myocardial infarction or hypovolemic shock—reduces peripheral blood flow and has been shown to increase PU risk by 2.8 times (21).

Conceptualizing pressure ulcers as chronic wounds allows for a more comprehensive understanding of their pathophysiology. Chronic cardiovascular conditions such as hypertension, dyslipidemia, and peripheral vascular disease contribute to sustained tissue hypoxia and impaired perfusion, forming the core of PU pathogenesis (22).

Multivariable analyses consistently identify cardiovascular diseases as independent risk factors for PU development (23, 24). These conditions lead to atherosclerotic plaque formation and vascular obstructions that impair peripheral circulation and increase localized ischemia. Conditions like heart failure further reduce cardiac output and exacerbate perfusion deficits, creating an ideal environment for ulcer formation.

Impaired perfusion delays wound healing and increases the risk of pressure-induced tissue injury. Therefore, maintaining cardiovascular integrity is essential for skin health, and preventive strategies should be prioritized in patients with known cardiac conditions (25).

Interestingly, while our study found a significant association between hypertension and PU development, the existing literature presents mixed results. Some studies support this link (26, 27), while others suggest the opposite (28). Chronic hypertension may lead to microvascular remodeling and rarefaction, reducing capillary surface area and increasing diffusion distance, thereby impairing tissue oxygenation (29).

Hypertension-induced microcirculatory damage may not only precipitate ulcer formation but also impair wound healing capacity. Arterial stiffness, endothelial dysfunction, and impaired vasodilation further exacerbate ischemic tissue injury. These hypotheses require support from biochemical and mechanistic studies (30).

From a clinical standpoint, ensuring hemodynamic stability is a key strategy in PU prevention. Optimizing tissue perfusion and controlling blood pressure can significantly reduce PU risk. Complementary strategies such as early mobilization and pressure-relieving support surfaces are also critical, especially for immobilized patients with cardiovascular comorbidities.

External pressure exceeding capillary pressure (~32 mmHg) impairs blood flow and leads to tissue hypoxia (8, 31). Clinical and experimental studies have shown that such ischemic damage can occur with as little as one to 4 h of continuous pressure (32). Thus, both intrinsic factors (e.g., comorbidities, hypoxia) and extrinsic factors (e.g., pressure load) must be addressed in comprehensive PU prevention strategies.

In conclusion, our analysis suggests that pressure ulcers typically develop in the presence of multiple comorbidities, which—although pathophysiologically distinct—converge via the common mechanism of hypoxia. Reduced perfusion and tissue oxygenation are as critical as immobility in determining PU risk.

Sedatives and the Risk of Pressure Ulcer Formation; Sedative and analgesic agents—such as benzodiazepines, propofol, and opioids—commonly used in surgical and palliative care settings may indirectly increase the risk of PU development. By reducing central nervous system activity, these drugs lower spontaneous movement and sensory perception, thereby prolonging periods of unrelieved pressure. In addition, sedatives blunt sympathetic vasoconstrictive responses and reduce systemic blood pressure, compromising microcirculatory perfusion. Several observational studies have demonstrated that prolonged or deep sedation correlates with early-onset ulcers, especially in patients with comorbid neurological or cardiovascular instability. Therefore, appropriate titration of sedative agents and scheduled repositioning should be considered integral parts of PU prevention (33).

Role of Vasopressors: Vasopressin and norepinephrine are widely used to maintain hemodynamic stability in critically ill or palliative patients, yet their vasoconstrictive effects may exacerbate tissue ischemia. Both agents reduce cutaneous and subcutaneous blood flow, diminishing capillary refill and oxygen delivery. Low doses of norepinephrine may sustain mean arterial pressure without harm, but prolonged or excessive administration can aggravate ischemic injury, accelerate early PU formation, and delay wound healing. Conversely, inadequate vasopressor support can lead to systemic hypotension and further hypoxia. Thus, balancing systemic perfusion with peripheral oxygenation through individualized vasopressor management is essential to minimize PU progression (34).

Corticosteroid Therapy and Ulcer Progression; Corticosteroids represent another pharmacologic factor influencing both the development and healing of pressure ulcers. Chronic corticosteroid use impairs fibroblast proliferation, collagen synthesis, and angiogenesis—processes vital for tissue repair. Patients receiving corticosteroids often exhibit skin thinning and decreased tensile strength, making them vulnerable to deeper and recurrent ulcers even under moderate pressure. Moreover, steroid-induced immunosuppression increases susceptibility to local infection and sepsis in ulcerated areas. Clinical data have shown delayed wound closure and higher recurrence rates among corticosteroid users, underscoring the need for intensified preventive care and nutritional support in this subgroup (35).

Taken together, the graphical representations (Figures 1–4) encapsulate the multifactorial nature of pressure-ulcer development—integrating demographic, systemic, and temporal dimensions into a coherent clinical framework. They also underscore that risk assessment must be dynamic: influenced not only by comorbid conditions but also by the timing and quality of preventive nursing practices during hospitalization.

Substance Abuse and Pressure Ulcer Risk; Substance abuse, particularly involving opioids or intravenous drugs, is an emerging but underrecognized contributor to PU development.

Drug abusers frequently suffer from malnutrition, dehydration, impaired hygiene, and prolonged immobilization. Repeated injection trauma and chronic vasoconstriction caused by stimulant or opioid use further compromise skin perfusion and healing capacity. Studies have shown that patients with substance abuse history exhibit significantly higher rates of chronic, non-healing pressure ulcers compared to non-abusers. Integrating addiction history and behavioral risk factors into comprehensive PU risk assessments may therefore improve early detection and prevention (36, 37). In essence, these figures do not merely complement the text; they visualize the story behind the data—how frailty, immobility, and systemic illness intersect to determine outcomes in real clinical settings.

## Conclusion

In this study, pressure ulcer development was found to be alarmingly prevalent among patients in a palliative care setting, underscoring the multifactorial nature of its pathogenesis. Independent risk factors such as neurological impairment, gastrointestinal dysfunction, cardiovascular comorbidities, prolonged hospitalization, and cognitive decline were all strongly associated with ulcer formation. Importantly, protective elements such as adherence to prevention protocols and complete care dependency highlight the critical role of structured, guideline-based nursing care. These findings suggest that comprehensive, multidisciplinary interventions targeting not only mobility but also systemic perfusion, nutritional status, and cognitive function are essential to mitigate PU risk in vulnerable populations. Integration of individualized risk assessment and proactive prevention strategies into routine care is strongly recommended to improve outcomes in palliative settings.

## Data Availability

The raw data supporting the conclusions of this article will be made available by the authors, without undue reservation.
